# Classifying office workers with and without cervicogenic headache or neck and shoulder pain using posture-based deep learning models: a multicenter retrospective study

**DOI:** 10.3389/fpain.2025.1614143

**Published:** 2025-07-07

**Authors:** Ui-jae Hwang, Junghun Han, Oh-yun Kwon, Yu Seong Chu, Sejung Yang

**Affiliations:** ^1^ Department of Rehabilitation Sciences, The Hong Kong Polytechnic University, Hong Kong SAR, China; ^2^ Department of Precision Medicine, Yonsei University Wonju College of Medicine, Wonju, Republic of Korea; ^3^ Department of Physical Therapy, College of Health Science, Laboratory of Kinetic Ergocise Based on Movement Analysis, Yonsei University, Wonju, Republic of Korea; ^4^ Department of Medical Informatics and Biostatistics, Graduate School, Yonsei University, Wonju, Republic of Korea

**Keywords:** deep learning, cervicogenic headache, neck pain, occupational diseases, posture

## Abstract

**Objective:**

To develop and evaluate deep learning models for classifying office workers with and without cervicogenic headache (CH) and/or neck and shoulder pain (NSP), based on habitual sitting posture images.

**Methods:**

This multicenter, retrospective, observational study analyzed 904 digital images of habitual sitting postures of 531 office workers. Three deep learning models (VGG19, ResNet50, and EfficientNet B5) were trained and evaluated to classify the CH, NSP, and combined CH + NSP. Model performance was assessed using 4-fold cross-validation with metrics including area under the curve (AUC), accuracy (ACC), sensitivity (Sen), specificity (Spe), and F1 score. Statistical significance was evaluated using 95% confidence intervals. Class Activation Mapping (CAM) was used to visualize the model focus areas.

**Results:**

Among 531 office workers (135 with CH, 365 with NSP, 108 with both conditions and 139 control group), ResNet50 achieved the highest performance for CH classification with an AUC of 0.782 (95% CI: 0.770–0.793) and an accuracy of 0.750 (95% CI: 0.731–0.768). NSP classification showed more modest results, with ResNet50 achieving an accuracy of 0.677 (95% CI: 0.640–0.713). In the combined CH + NSP classification, EfficientNet B5 demonstrated the highest AUC of 0.744 (95% CI: 0.647–0.841). CAM analysis revealed distinct focus areas for each condition: the cervical region for CH, the lower body for NSP, and broader neck and trunk regions for combined CH + NSP.

**Conclusion:**

Deep learning models show potential for classifying CH and NSP based on habitual sitting posture images, with varying performances across conditions. The ability of these models to detect subtle postural patterns associated with different musculoskeletal conditions suggests their possible applications for early detection and intervention. However, the complex relationship between static posture and musculoskeletal pain underscores the need for a multimodal assessment approach in clinical practice.

## 1 Introduction

Office workers spend an average of five to six hours daily in sedentary positions, with computer usage exceeding three hours per day significantly correlating with increased musculoskeletal discomfort in the neck, head, and upper extremities (1, 2). Poor ergonomic practices, particularly habitual slumped sitting postures, are associated with increased posterior pelvic rotation, thoracic flexion, and forward head posture, leading to biomechanical stress and spinal tissue dysfunction (3–6).

Postural deviations have been specifically linked to cervicogenic headaches (CH), where continuous nociceptive input from upper cervical structures sensitizes the trigeminocervical complex, increasing pain sensitivity (7–9). Importantly, musculoskeletal dysfunctions are present not only in cervicogenic headaches but also in primary headache disorders such as migraine, where cervical impairments contribute to both peripheral and central sensitization mechanisms (10, 11). Similarly, sustained awkward postures and prolonged computer use are recognized risk factors for neck and shoulder pain (NSP), with affected individuals exhibiting altered muscle activity and difficulty maintaining upright posture (12–19). Recent advances in deep learning have demonstrated remarkable success in medical image analysis, achieving performance comparable to practicing radiologists in various diagnostic tasks (20, 21).

Despite the established associations between posture and musculoskeletal pain, current assessment methods rely primarily on subjective clinical evaluation or expensive motion analysis systems. There is a significant gap in automated, objective tools for classifying individuals at risk of CH and NSP based on easily obtainable postural data. While deep learning has revolutionized medical imaging, its application to posture-based classification of musculoskeletal conditions remains largely unexplored. Furthermore, the complex relationship between static posture and pain conditions requires sophisticated pattern recognition capabilities that traditional assessment methods cannot provide.

The recent shift toward remote work environments has intensified the need for accessible screening tools, as workers may lack ergonomically optimized setups, increasing musculoskeletal disorder risk (22). Early identification of at-risk individuals is crucial for implementing preventive interventions and reducing the burden of chronic pain conditions. Deep learning models offer the potential to detect postural patterns invisible to human observers, providing an objective, scalable solution for workplace health screening. Therefore, this study addresses the specific research gap in automated posture-based screening tools for occupational musculoskeletal disorders.

The purpose of the present study is (1) to develop and evaluate deep learning models (VGG19, ResNet50, and EfficientNet B5) for classifying office workers with and without CH and/or NSP based on habitual sitting posture images and (2) to identify specific postural regions that contribute to the classification of different musculoskeletal conditions using Class Activation Mapping analysis. We hypothesize that these deep learning models will accurately differentiate affected individuals from healthy controls, with distinct postural patterns being identifiable for each condition, thereby offering a novel automated approach for early detection in occupational health settings.

## 2 Methods

### 2.1 Study design and participants

This multicenter retrospective observational study was conducted in accordance with the Strengthening the Reporting of Observational Studies in Epidemiology (STROBE) guidelines for case-control studies. Digital images of habitual sitting for office workers (OWs) obtained from musculoskeletal screening tests to prevent industrial accidents were used to examine the risk factors for musculoskeletal disorders in 11 public service offices from April 2021 to February 2023. The OW data generated from musculoskeletal screening tests to prevent industrial accidents were obtained by visiting musculoskeletal healthcare programs in 11 public service offices. The Institutional Review Board of the Yonsei University Mirae Campus waived the requirement for informed consent before analysis, as the study used data already acquired by musculoskeletal screening tests to prevent industrial accidents. Personal information was not obtained to protect participants' anonymity. In total, 531 OWs were screened for eligibility (Table 1). Sample size calculation was based on achieving 80% power to detect a medium effect size (Cohen's *d* = 0.5) with *α* = 0.05 for binary classification tasks. With an expected prevalence of 25% for cervicogenic headache and 70% for neck and shoulder pain in office worker populations, a minimum sample of 500 participants was determined necessary. The final sample of 531 participants exceeded this requirement, ensuring adequate statistical power. OWs who had been using computers in the office for more than two years were screened. Individuals with CH were included if their pattern of symptoms was consistent with the diagnostic criteria of the CH International Study Group (23); (score 1) precipitation of head pain, similar to the usually occurring one-by-neck movement or sustained awkward head positioning, or both, and by external pressure over the cervical or occipital region on the symptomatic side; (score 2) restriction of the range of motion in the neck; and (score 3) unilateral head pain without side shift. Individuals with NSP were included if they (score 1) reported NSP (e.g., upper trapezius, levator scapula, or lower cervical pain) intensity over the last month as greater than 3 of 10 on a Numerical Rating Scale (NRS), (score 2) had a history of NSP for more than one month and (score 3) had no history of cervicogenic headache. Individuals without CH or NSP were eligible if they had no history or experience of neck pain in the last three months. The exclusion criteria for OWs with and without CH and NSP were diagnosed with hypertension, rheumatological conditions, or a history of spinal surgery.

**Table 1:** Participants characteristics.

Variables	CH^a^ (*N* = 135)	NSP^b^ (*N* = 365)	CH + NSP (*N* = 108)	Healthy (*N* = 139)
Sex (Male; Female)	M = 10; F = 125	M = 29; F = 336	M = 10; F = 98	M = 22; F = 117
Age (Mean ± SD)	35.7 ± 8.0	36.1 ± 7.4	35.7 ± 8.0	36.5 ± 7.9
Work duration (Mean ± SD)	9.3 ± 7.2	10.1 ± 7.4	9.3 ± 7.5	10.3 ± 7.5
VAS^c^ (Mean ± SD)	5.8 ± 1.7	5.8 ± 1.5	6.2 ± 1.7	0.0 ± 0.3

^a^ CH, cervicogenic headache.

^b^ NSP, neck and shoulder pain.

^c^ VAS, visual analog scale.

### 2.2 Habitual sitting posture using two-dimensional digital image analysis

A smartphone equipped with video recording capabilities (4 K resolution, 3,840 × 2,160 pixels at 60 frames per second) was secured on a tripod positioned 100 cm from the side of the chair with its height adjusted to align with the level of the participant's ear tragus. The participants were seated on an adjustable stool without back support, positioned such that the height aligned with their popliteal crease, ensuring a 90-degree angle at both the hips and knees, with the feet in a neutral plantar grade position. In the first photograph, the subjects were instructed to adopt a comfortable and habitual sitting posture, refrain from adjusting their position in the seat, breathe normally, and look forward. The second photograph was captured after standing up and sitting back down in a comfortable position.

### 2.3 Deep learning models

**Figure 1:**
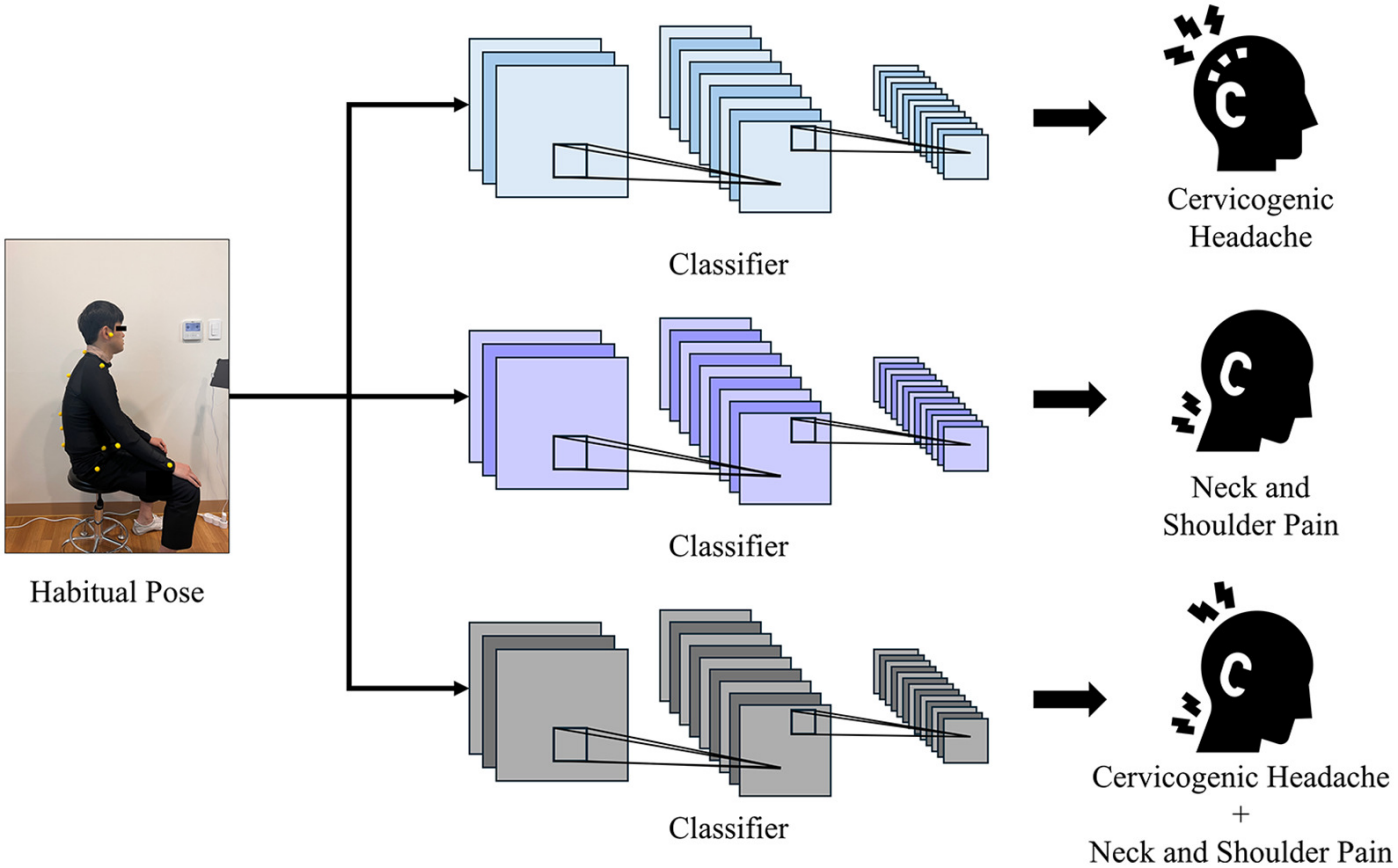
Overall workflow.

The deep learning models used for the performance comparison were the VGG19 (24), ResNet50 (25), and EfficientNet (26) models, which representatively selected and compared models that showed excellent performance in classification. We conducted individual training and comparisons of the classification tasks for the CH, NSP, and CH + NSP using each model. The workflow is illustrated in Figure 1.

VGG19 is a deep convolutional neural network consisting of 19 layers that learn deep features using small 3 × 3 filters. After each convolutional block, a 2 × 2 max pooling layer was placed, which ultimately performed image classification using three fully connected layers. VGG19 has a relatively simple network structure, making it easy to understand and implement. The consistent use of 3 × 3 convolutional filters reduced the complexity and increased the network depth to improve performance.

ResNet50 is a deep convolutional neural network consisting of 50 layers that effectively increases the network depth through residual learning. ResNet50 introduces residual blocks to solve the gradient loss problem even in deep networks where learning is difficult. Each residual block maintains smooth information flow by adding input directly to the output through a skip connection. ResNet50 performs well in various computer vision tasks such as image classification and object detection and exhibits efficient learning and generalization capabilities. Moreover, it is effective for transfer learning using pretrained weights.

EfficientNet is a type of convolutional neural network that systematically scales up the width, depth, and resolution of a network to achieve higher performance while maintaining computational efficiency. EfficientNet models, such as EfficientNet B0 to B7, utilize a compound scaling method that uniformly scales all network dimensions based on a fixed set of scaling coefficients. This approach allows EfficientNet to effectively balance the tradeoffs between accuracy and computational resources. EfficientNet enhances feature extraction while reducing the number of parameters and Floating Point Operations (FLOPs) by employing a novel architecture called the MBConv block, which combines depthwise separable convolutions and squeeze-and-excitation modules, EfficientNet enhances feature extraction while reducing the number of parameters and FLOPs. The EfficientNet models achieved state-of-the-art performance on various image classification benchmarks and exhibited excellent transfer learning capabilities with pretrained weights. Their efficiency and high accuracy make them suitable for deployment in resource-constrained environments such as mobile devices and edge computing applications.

### 2.4 Explanatory deep learning model

To ensure the reliability of the deep learning model and the basis for this study, the model was verified using a representative class activation mapping (CAM) among the explainable method. CAM is a technique for visualizing deep learning models that focus on image classification. It combines the feature map and class weights of the last convolutional layer to generate class-specific activation maps. This clarifies the predictive basis of the model and improved its explanatory ability. CAM are used to improve the transparency of models in various fields, such as medical image analysis and autonomous driving. This technique is particularly useful for understanding the inherent decision-making processes of complex deep-learning models.

## 3 Results

### 3.1 Datasets

**Figure 2:**
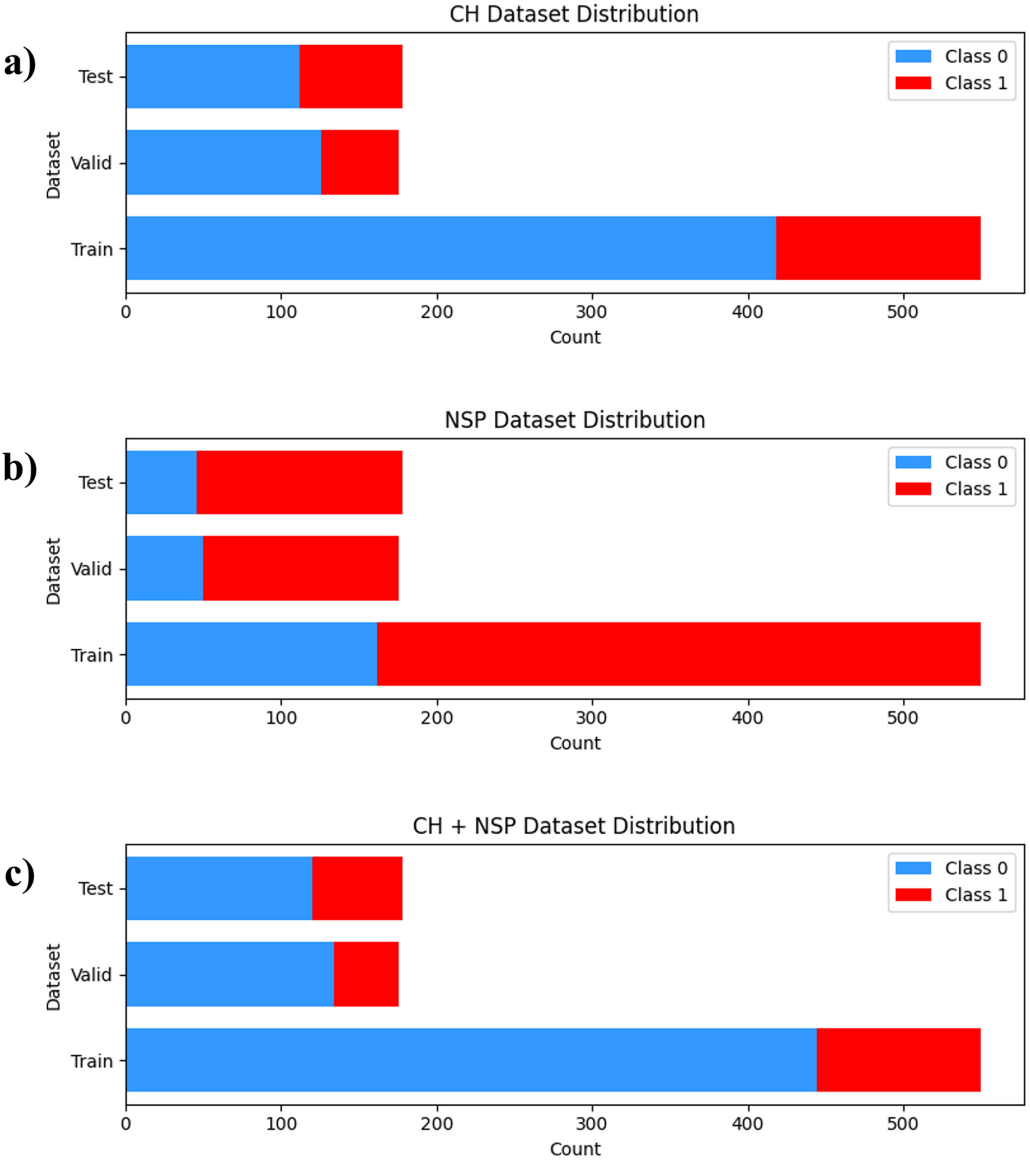
Distribution of each dataset. **(a)** CH dataset **(b)** NSP dataset **(c)** CH + NSP dataset.

The cervicogenic headache (CH) and neck and shoulder pain (NSP) of each subject were measured using a survey, and 904 images of 531 subjects sitting in habitual postures on chairs were collected. Data from 331 participants were used as training data, 100 as validation data, and 100 as test data, comprising 550 training, 176 validation, and 178 test images. Among the 531 participants, 135 had CH and 365 had NSP. In addition, 108 participants had both CH and NSP. The detailed composition of each dataset is shown in Figure 2.

### 3.2 Model performance

The results of classifying the normal, CH, NSP, and CH + NSP groups based on habitual postures are shown in Table 2. The results were obtained through 4-fold cross-validation, with the numbers in parentheses representing 95% confidence intervals. The bold text indicates the highest value for each target. Comparisons were performed using VGG19, ResNet50, and EfficientNet B5. In the CH group, ResNet50 achieved superior performance with an AUC of 0.7821 (95% CI: 0.7703–0.7939) and accuracy of 75.00% (95% CI: 73.14–76.86), outperforming VGG19 (AUC: 0.7263) and EfficientNet B5 (AUC: 0.6371). NSP classification demonstrated lower discriminative capacity, with ResNet50 attaining 67.70% accuracy (95% CI: 64.01–71.38) and VGG19 showing marginally higher specificity (32.61% vs. 26.09% in ResNet50). For combined cervicogenic headache and neck/shoulder pain (CH + NSP) classification, EfficientNet B5 demonstrated superior performance with an AUC of 0.7443 (95% CI: 0.6475–0.8410) and accuracy of 73.74% (95% CI: 66.07–81.40). ResNet50 showed marginally lower performance, achieving an AUC of 0.7348 (95% CI: 0.7228–0.7468) and accuracy of 77.11% (95% CI: 75.12–79.09). The models exhibited moderate sensitivity (ResNet50: 34.91%, EfficientNet B5: 41.38%) but high specificity (ResNet50: 97.50%, EfficientNet B5: 89.37%), indicating stronger performance in identifying true negatives than true positives.

**Table 2:** Classification results.

Target	Model	AUC^a^	Accuracy	Sensitivity	Specificity	F1 Score
CH^b^	ResNet50	**0.782** (0.770, 0.793)	**0.750** (0.731, 0.768)	0.492 (0.385, 0.599)	**0.902** (0.854, 0.950)	**0.591** (0.521, 0.662)
	EfficientNet B5	0.637 (0.522, 0.751)	0.676 (0.601, 0.753)	**0.601** (0.572, 0.657)	0.890 (0.848, 0.933)	0.416 (0.249, 0.583)
	VGG19	0.726 (0.640, 0.812)	0.716 (0.661, 0.771)	0.435 (0.356, 0.515)	0.881 (0.788, 0.974)	0.532 (0.459, 0.605)
NSP^c^	ResNet50	0.611 (0.575, 0.646)	**0.677** (0.640, 0.714)	**0.822** (0.710, 0.934)	0.261 (0.080, 0.441)	**0.790** (0.748, 0.831)
	EfficientNet B5	0.527 (0.375, 0.680)	0.622 (0.521, 0.723)	0.729 (0.590, 0.868)	0.315 (0.184, 0.446)	0.739 (0.655, 0.823)
	VGG19	**0.612** (0.588, 0.634)	0.674 (0.578, 0.770)	0.795 (0.678, 0.913)	**0.326** (0.286, 0.366)	0.782 (0.706, 0.858)
CH + NSP	ResNet50	0.734 (0.722, 0.746)	**0.771** (0.751, 0.791)	0.349 (0.281, 0.418)	**0.975** (0.936, 1.000)	0.497 (0.442, 0.552)
	EfficientNet B5	**0.744** (0.647, 0.841)	0.737 (0.660, 0.814)	**0.414** (0.321, 0.506)	0.893 (0.799, 0.988)	**0.508** (0.396, 0.619)
	VGG19	0.691 (0.668, 0.714)	0.716 (0.688, 0.744)	0.371 (0.313, 0.427)	0.883 (0.854, 0.912)	0.459 (0.401, 0.517)

The bold text indicates the highest value for each target.

^a^ AUC, area under the receiver operating characteristic curve.

^b^ CH, cervicogenic headache.

^c^ NSP, neck and shoulder pain.

**Figure 3:**
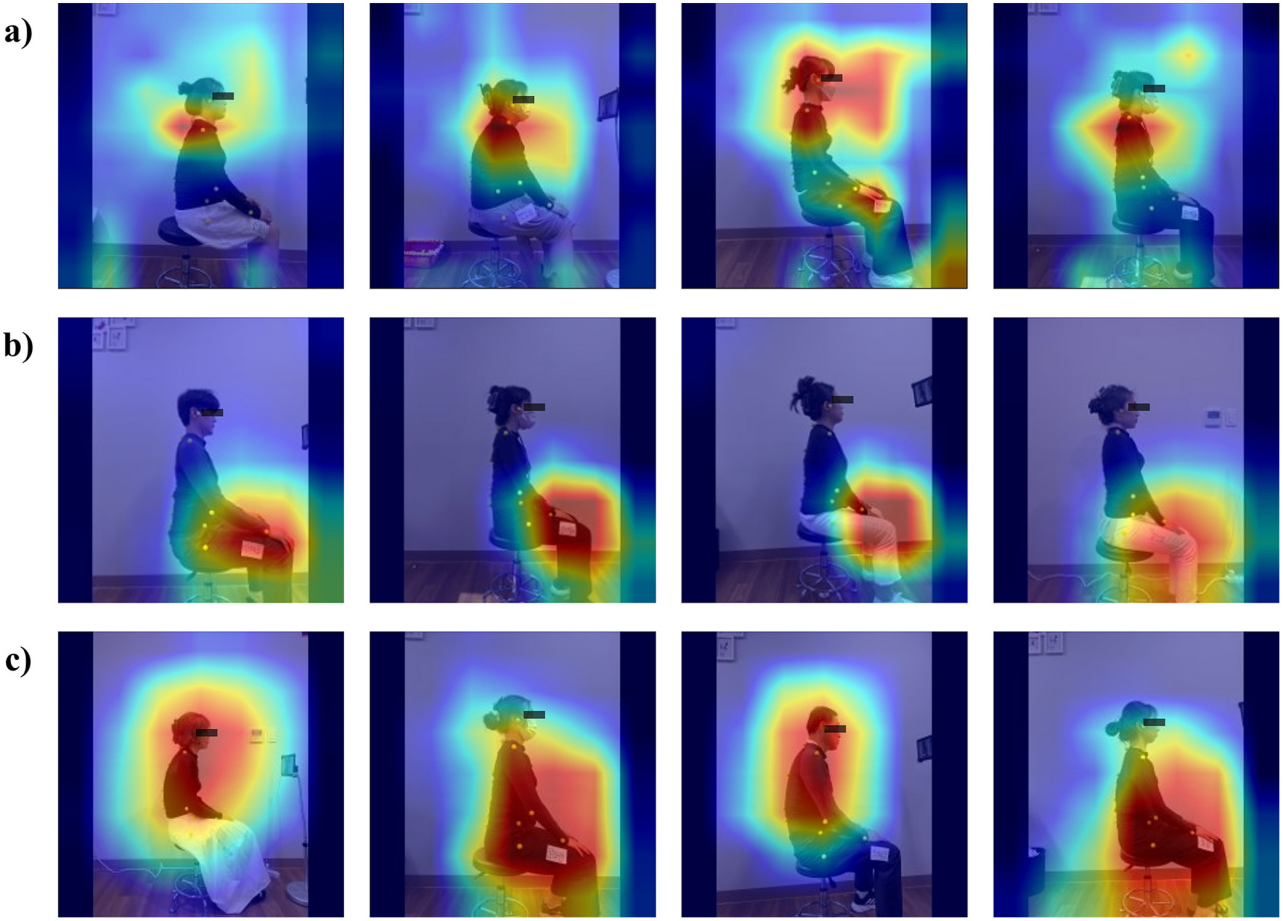
CAM results. **(a)** CH CAM **(b)** NSP CAM **(c)** CH + NSP CAM.

Figure 3 shows the CAM for each task, illustrating that different areas were activated for each specified task. Each CAM is represented by adopting the best-performing ResNet50 model. As shown in Figure 3a, for CH classification, activation was observed around the head and neck. As shown in Figure 3b, during NSP classification, activation was observed around the knee area. Additionally, as shown in Figure 3c, classifying both the CH and NSP showed that activation was evenly distributed throughout the body.

## 4 Discussion

This study demonstrates the feasibility of using deep learning models to classify office workers with cervicogenic headache (CH) and neck and shoulder pain (NSP) based on habitual sitting posture images. Our findings reveal distinct classification performances across conditions: CH classification achieved the highest accuracy with ResNet50, NSP classification showed more modest results, while combined CH + NSP classification demonstrated intermediate performance. Class activation mapping (CAM) analysis revealed condition-specific postural patterns, with models focusing on the cervical region for CH, lower body regions for NSP, and broader neck-trunk areas for combined conditions.

Our CH classification results align with established research demonstrating postural differences in cervicogenic headache patients. The CAM analysis revealed that models focused primarily on the cervical spine region when classifying CH cases, which aligns with the understanding that CH originates from the cervical structures and is associated with upper cervical spine dysfunction (9). This finding is consistent with previous research showing that patients with CH exhibit a greater forward head posture than asymptomatic individuals (7). Mingels et al. found that individuals with posture-induced headaches demonstrated increased passive head protraction and habitual forward head position further from the end range compared with controls (27). While our study did not directly measure end-range positions, the attention to cervical posture using the deep learning model suggests that even subtle postural deviations detectable in images may be indicative of the underlying cervical dysfunctions associated with CH.

The modest performance in NSP classification reflects the complex, multifactorial nature of neck and shoulder pain documented in the literature. From a physical therapy perspective, NSP is influenced not only by posture, but also by factors such as muscle activity, strength, endurance, and psychosocial variables (28). Our finding that models focused on lower body regions for NSP classification interestingly aligns with the findings of Straker et al. showing a relationship between prolonged NSP and sitting spinal posture, particularly in the lumbar region (29). The focus on lower body posture for NSP classification suggests that pelvic tilt and lower limb alignment alterations may play a role in NSP development or persistence. However, the lower accuracy of NSP classification compared with that of CH indicates that postural factors alone do not fully capture the complexity of NSP.

The ability of deep learning models to detect subtle postural patterns offers significant potential for early screening in occupational health settings. For clinicians, these findings suggest that automated postural assessment could complement traditional evaluation methods, particularly in resource-limited environments or large-scale workplace screenings. The condition-specific focus patterns identified through CAM analysis provide objective guidance for targeted interventions.

For the combined CH + NSP classification, the results indicated an intermediate performance between the CH-only and NSP-only tasks. CAM analysis revealed that the models focused on a broader region from the neck and trunk for this combined condition. This suggests that the presence of both conditions may manifest as detectable postural patterns across a larger portion of the spine, but with less specificity than CH alone. Clinically, this highlights the potential overlap and interactions between CH and NSP that can present diagnostic and therapeutic challenges. The broader focus of the model on the neck and thoracic regions for combined CH + NSP cases supports the idea that postural and muscular interactions between these areas may contribute to the co-occurrence of these conditions. This aligns with the findings of Caneiro et al. who demonstrated that different sitting postures influence both cervical spine posture and muscular activity of the cervicothoracic region (30).

However, our findings underscore the importance of integrating postural assessment with other key clinical measures. Future clinical applications should correlate these automated postural classifications with established outcomes such as cervical range of motion and patient-reported subjective perceptions, as these represent critical components of comprehensive musculoskeletal assessment. The reliability and validity of cervical range of motion measurements have been well-established across different assessment devices and populations, demonstrating their importance as objective clinical outcomes in patients with neck pain conditions (31). Additionally, patient subjective perceptions and experiences provide crucial insights into the real-world impact of musculoskeletal conditions that may not be captured through objective measures alone. Understanding why patient experience measures are essential in physical therapy practice can enhance clinical effectiveness and provide excellent patient-centered care delivery (32). The integration of objective postural data with functional measures such as cervical range of motion and patient-centered outcomes could enhance both diagnostic accuracy and treatment planning, creating a more holistic approach to musculoskeletal assessment that addresses both biomechanical factors and patient experiences.

Several limitations must be acknowledged. First, our study relied on static posture assessment from 2D images, which may not capture the dynamic aspects of postural behavior during prolonged computer work. Static postural assessment alone may be insufficient to fully characterize NSP, which often involves complex interactions among multiple factors. Second, the cross-sectional design prevents determination of causal relationships between posture and pain conditions. Third, the modest performance in NSP classification suggests that postural factors alone may be insufficient for accurate identification of this complex condition, reinforcing the need for multimodal assessment approaches in clinical practice to evaluate patients with neck and shoulder complaints. From a technical perspective, the lower performance metrics for the NSP classification suggest that the postural features extracted by the models are less distinctly associated with the NSP than with the CH. The models may detect some posture-related patterns; however, they appear to have limited specificity in differentiating NSP from asymptomatic cases. The overall low specificity observed in our results indicates the need for improvement in model development and training strategies. Fourth, our study was conducted in a specific population of Korean office workers, which may limit generalizability to other demographic groups or work environments. Additionally, the deep learning models require standardized image acquisition protocols, which may limit their practical implementation in diverse workplace settings. The inter-observer reliability of pain condition classification and the potential for selection bias in our retrospective design represent additional methodological considerations.

The applicability of our findings extends beyond the immediate study population, as prolonged computer use and poor workplace ergonomics represent global occupational health challenges. However, successful translation to clinical practice requires validation across diverse populations, work environments, and cultural contexts. The standardized image acquisition protocol developed in this study could facilitate multi-center validation studies. From a technical standpoint, our findings demonstrate varying model performance across different conditions and architectures. In the CH classification task, ResNet50 demonstrated superior performance with high specificity, which is crucial for reducing false positives. For the NSP classification task, both ResNet50 and VGG19 outperformed EfficientNet B5, with notably high sensitivity but low specificity across all models. For the CH + NSP classification task, ResNet50 and EfficientNet B5 excelled, indicating the potential of these architectures for multiclass classification involving both conditions. For widespread implementation, future research should investigate the model performance across different age groups, work settings, and ergonomic conditions. Additionally, the development of mobile-based applications could enhance accessibility and enable real-time postural monitoring in various occupational contexts.

Future research should adopt longitudinal designs to establish temporal relationships between postural patterns and pain development. Integration of additional data modalities, such as electromyographic activity, functional movement assessments, and psychosocial factors, could improve classification accuracy, particularly for complex conditions like NSP. Future studies incorporating additional data modalities such as electromyogram or pain provocation tests may improve the classification of these complex combined presentations. The development of dynamic postural assessment capabilities through video analysis represents a promising avenue for capturing more comprehensive postural behaviors. Furthermore, investigation of intervention effectiveness guided by automated postural assessment could demonstrate the clinical utility of these tools in preventing and managing occupational musculoskeletal disorders.

## 5 Conclusion

This study demonstrates the potential of deep learning models for classifying the cervicogenic headache (CH) and neck and shoulder pain (NSP) and their combinations based on habitual sitting posture images. For CH classification, ResNet50 achieved the highest accuracy of 75% and an AUC of 0.7821, whereas the NSP classification showed a more modest performance with 67.70% accuracy. The combined CH + NSP classification achieved intermediate results, with EfficientNet B5 reaching an AUC of 0.744. Class activation mapping (CAM) analysis revealed distinct areas of focus: the cervical region for CH, lower body for NSP, and broader neck and trunk regions for combined CH + NSP. These findings suggest that deep-learning models can detect subtle postural patterns associated with different musculoskeletal conditions, potentially offering a valuable tool for early detection and intervention in clinical settings. However, varying performance across conditions underscores the complex relationship between static posture and musculoskeletal pain, and highlights the need for multimodal assessment approaches in clinical practice.

## Data Availability

The raw data supporting the conclusions of this article will be made available by the authors, without undue reservation.
